# Integrated Role of Nutrition and Physical Activity for Lifelong Health

**DOI:** 10.3390/nu11071437

**Published:** 2019-06-26

**Authors:** Karsten Koehler, Clemens Drenowatz

**Affiliations:** 1 Department of Sport and Health Sciences, Technical University of Munich, 80992 München, Germany; 2 Department of Nutrition and Health Sciences, University of Nebraska-Lincoln, Lincoln, NE 68583, USA; 3 Division of Physical Education, University of Education Upper Austria, 4020 Linz, Austria

It is well established that healthy nutrition and physical activity (PA) are key lifestyle factors that modulate lifelong health through their ability to improve body composition, musculoskeletal health, and physical and cognitive performance, as well as to prevent metabolic diseases including obesity, diabetes mellitus, and cardiovascular disease across the lifespan. While the health benefits of nutrition and PA are often studied singularly, it has become more and more evident that the integration of nutrition and PA has the potential to produce greater benefits when compared to strategies focusing solely on one or the other. This Special Issue entitled “Integrated Role of Nutrition and Physical Activity for Lifelong Health” is devoted to manuscripts that highlight this integrational approach on various outcomes related to lifelong health. In response to our call, a total of 14 manuscripts were included. In addition to research focusing on the integrated benefits of nutrition and PA on various markers related to health and performance across a broad spectrum of life stages, several studies examining how PA has the potential to change food consumption were also included.

The featured article by Gustafson et al. reports that food choices are altered in the context of exercise. In this experiment, gym goers were asked to choose between a healthy and unhealthy snack to be consumed after completion of their exercise. Compared to when this choice was made prior to exercise, individuals after completing exercise were less likely to choose a healthy snack and instead chose the unhealthy option [1]. This possibly unhealthy impact of sport participation on food choices was extended in another study by Koenigstorfer, although participation was limited to passive viewership in this instance. Regardless, visits to sporting events were associated with an increased preference for unhealthy food items, although it was notable that a similar increase was also observed for some non-sport related venues, such as music concerts [2]. In a study focusing on the health effects of dietary choices in the context of an exercising population, Wirnizter et al. report that vegetarian and vegan athletes exhibited lower body weights and vegetarian athletes reported a lower prevalence of allergies when compared to their omnivore counterparts, while many other health-related outcomes were similar. These findings suggest that adherence to a vegetarian or even vegan diet is not detrimental to the health of endurance athletes [3]. 

Two studies [4,5] examine the role of diet and PA on health-related parameters in youth. Drenowatz et al. show that healthy dietary choices and sports participation are independently associated with motor competence, which is an important contributor to an active and healthy lifestyle [6,7]. Meng et al. report beneficial effects of an education-based obesity prevention program on dietary intake in adolescents. This study also highlights the importance of club sports participation, as PA levels were higher during the sport season compared to the off-season [5]. The promotion of leisure time PA outside-specific club settings thus remains a critical component for future research.

The importance of an active and healthy lifestyle is also shown by Van Elten et al. who examine the sustainability of a diet and PA intervention on cardiometabolic health in women. Even though a potential effect of snack intake on insulin resistance 3 to 8 years after the intervention was shown, the results emphasize the importance of current lifestyle choices for cardio-metabolic health. In order to achieve sustainable lifestyle changes, prolonged engagement in the intervention may be necessary [8]. A review by Balan et al. further emphasizes the importance of PA and diet to counteract age-related diseases by showing beneficial effects of fiber and unsaturated lipids on telomere health. While the authors acknowledge that more research is needed, they also suggest a protective effect of PA on telomere maintenance, which contributes to health in old age [9].

Given the importance of healthy lifestyle choices, a key question remains on how to promote healthy dietary choices and PA among a broad population. Electronic health (Ehealth) approaches could provide economically feasible opportunities—Doorn-van-Atten et al. examine the efficacy of an eHealth intervention in older adults, who are commonly less receptive to technology-based intervention strategies. Their results indicate beneficial effects of PA self-monitoring along with educational materials on lifestyle choices [10]. Educational materials were also shown to improve self-efficacy to engage in PA and consume a healthy diet in women as well as to provide growth-promoting animal protein to their stunted offspring [11].

In addition to the improvement of health outcomes, three articles address the role of nutrition and PA in modulating inflammation, which is associated with chronic disease and aging. When comparing elderly individuals across the PA spectrum, Ferrer et al. report that active individuals not only exhibited improved body composition but also improved blood profiles of inflammatory and anti-inflammatory markers [12]. These findings are strongly supported by Draganidis et al. who compared elderly individuals with low vs. high levels of systemic inflammation and found that low systemic inflammation is associated with greater levels of PA, particularly moderate-to-vigorous PA as well as increased antioxidant intake [13]. Although inflammation was not found to be significantly affected in a study assessing the impact of a purified vegan diet in exercising rats, this diet—especially when combined with an exercise regimen—was associated with improved body composition, metabolic markers, and physical performance [14]. 

Another topic in the area of exercise nutrition that is heavily debated involves protein requirements for exercisers. Isenmann et al. demonstrate the importance of adequate protein and carbohydrate intake from foodstuffs following an exercise bout for the facilitation of muscle regeneration while minimizing the inflammatory response [15]. Reckmann et al. present a novel method for quantifying exogenous protein oxidation using a breath test. While their findings failed to show alterations in whole-body metabolism in response to short-term fluctuations in protein intake, their data suggest that there is large inter-individual variability in response to protein-restricted diets. Accordingly, further research is needed to clarify the influence of dietary choices and nutrient intake on protein metabolism in active populations [16].

Taken together, the research presented in this Special Issue supports the previously emphasized role of integrating diet and PA on general health and well-being across the lifespan. A key issue for future research, therefore, will be the implementation of intervention strategies that promote an active and healthy lifestyle along with the exploration of the specific mechanisms that explain the individual and combined contribution of PA and nutrition to various health outcomes, including cardiovascular and metabolic diseases as well as orthopedic problems and depression.
